# A comparative study of conventional, High-flow, and jet ventilation via the Wei nasal jet tube for oxygen therapy in patients undergoing bronchoscopic intervention under deep sedation: a randomized controlled trial

**DOI:** 10.3389/fmed.2026.1820327

**Published:** 2026-06-24

**Authors:** Ai-Di Zhang, Xiang Ge, Xiao-Li Li, Qing-Hao Cheng, Ming-Yuan Yang

**Affiliations:** 1 Department of Anaesthesiology, Emergency General Hospital, Beijing, China; 2 Department of Respiratory, Emergency General Hospital, Beijing, China

**Keywords:** bronchoscopic intervention, conventional oxygen therapy, high-flow nasal cannula, supraglottic jet oxygenation and ventilation, Wei nasal jet tube

## Abstract

**Background:**

Hypoxemia is a frequent complication in the bronchoscopic interventions (BI) under deep sedation due to shared airway challenges. The Wei nasal jet tube (WNJ) enables supraglottic jet oxygenation and ventilation (SJOV) without tracheal intubation, but its efficacy and safety compared to conventional oxygen therapy (COT) or high-flow nasal cannula (HFNC) in patients with mild-to-moderate airway stenosis remain unclear.

**Methods:**

In the prospective randomized controlled trial, 150 patients through BI under deep sedation were allocated to three groups: COT (fractional inspired oxygen (FiO_2_ 1.0, 10 L/min), HFNC (FiO_2_ 1.0, 40 L/min), or SJOV via WNJ (FiO_2_ 1.0, driving pressure 0.6 bar, respiratory rate 600 cycles/min). Primary outcomes included the incidence of intraoperative hypoxemia (SpO_2_ < 90%), severe hypoxemia (SpO_2_ < 80%), the requirement for mask positive-pressure oxygenation (MPPO) and endotracheal intubation. Arterial blood gas parameters, hemodynamic variables, adverse events and procedure duration were also analyzed.

**Results:**

A sum of 150 patients participated in the study. The incidences of hypoxemia, severe hypoxemia, and MPPO were compared among the three groups. The overall incidence of hypoxemia differed significantly (*P* = 0.020), with the SJOV group showing a lower rate than the COT group (*P* = 0.018). The overall incidence of severe hypoxemia differed significantly among groups (*P* = 0.024), SJOV showed a notably reduced incidence compared to COT (*P* = 0.016). MPPO incidence varied significantly among groups (*P* = 0.022). SJOV showed a lower rate than COT (*P* = 0.014). PaO_2_ was significantly higher in the SJOV group compared to both the COT and HFNC groups (*P* < 0.001 for both). The SJOV group exhibited significantly lower lactic acid levels than both the COT group (*P* < 0.001) and the HFNC group (*P* = 0.006). No significant differences were observed in endotracheal intubation, PaCO_2_, pH, hemodynamic stability, total propofol and remifentanil consumption, adverse events or procedure duration among groups.

**Conclusion:**

SJOV delivered via the Wei nasal jet tube improved intraoperative oxygenation and reduced hypoxemia compared with COT during bronchoscopic intervention under deep sedation, without increasing adverse events. These findings support SJOV as a feasible oxygenation and ventilation strategy for selected patients with mild-to-moderate airway stenosis.

**Clinical trial registration:**

https://www.chictr.org.cn/searchproj.html, identifier ChiCTR2100054123.

## Background

Maintaining adequate oxygenation through rigid bronchoscopy in patients with mild-to-moderate airway stenosis is particularly challenging because of the shared airway, reduced airway patency, and deep sedation induced respiratory depression (1, 2). Conventional oxygen therapy (COT), delivered through low-flow nasal cannula, is often inadequate in the setting and is linked to a higher occurrence of hypoxemia (3, 4). High-flow nasal cannula (HFNC) has become the current commonly used noninvasive oxygenation strategy, creating a positive end-expiratory pressure (PEEP), a comparable effect for enhancing the state of oxygenation (5). However, it does not provide active ventilation and may be insufficient when ventilation perfusion mismatch and carbon dioxide (CO_2_) retention develop during prolonged or complex airway interventions (6).

Supraglottic jet oxygenation and ventilation (SJOV) represents a fundamentally different approach (7). By delivering pressurized oxygen pulses through a supraglottic conduit, SJOV actively ventilates the lungs, generates intrinsic PEEP, and enhances CO_2_ washout. Among these, the Wei nasal jet tube (WNJ) stands out for its ease of use, good patient tolerance and compatibility with bronchoscopic manipulation; its nasopharyngeal placement mitigates laryngeal irritation while enabling the simultaneous delivery of oxygen and ventilatory support, thus making it well suited for bronchoscopic intervention (8, 9).

Therefore, the study conducted a randomized controlled trial to compare COT, HFNC, and SJOV during bronchoscopy in patients with mild to moderate airway stenosis, testing the hypothesis that SJOV would provide superior oxygenation and reduce hypoxemia compared with both COT and HFNC.

## Methods

### Trial design

This randomized controlled trial took place at a single-center in Emergency General Hospital, Beijing, China. This study received ethical approval from the hospital’s Medical Ethics Committee (Approval Number: K21-39e1), and was prospectively registered in the Chinese Clinical Trial Registry (Registration Number: ChiCTR2100054123) on December 9, 2021. No interim analyses were planned or conducted during the trial. No protocol modifications were implemented after trial initiation that affected patient enrollment, sedation protocol, oxygenation strategy, rescue criteria, or outcome definition. Each participant gave their written informed consent.

### Participants

Patients scheduled to undergo bronchoscopy airway intervention in the Respiratory interventional therapy Center of the Emergency General Hospital were considered for inclusion from July 2022 to July 2024. Inclusion criteria: (1) The age of participants varied between 18 and 80; (2) American Society of Anesthesiologists (ASA) classification II-III; (3) No severe nasopharyngeal diseases, such as severe nasal septum deviation or nasopharyngeal tumors that would interfere with WNJ insertion; (4) Central airway stenosis of less than 70% as assessed by preoperative computed tomography (CT). Exclusion criteria: (1) Hypertension and tachycardia; (2) Comatose state (Glasgow Coma Scale score < 8) or inability to cooperate with the procedure; (3) Preexisting tracheotomy, endotracheal intubation, or other airway devices that would conflict with WNJ tube placement; (4) Admission peripheral oxygen saturation (SpO_2_) below 90% or arterial oxygen partial pressure (PaO_2_) less than 60 mmHg (measured on room air); (5) Severe airway stenosis (more than 70% luminal obstruction confirmed by CT); (6) History of severe psychiatric diseases or neurological disorders that could affect anesthetic recovery; (7) History of sedative-hypnotic use, or alcohol abuse; (8) Intraoperative need for changing the anesthesia method; (9) Intraoperative massive bleeding, which would obscure airway visualization and interfere with WNJ management.

### Randomization and blinding

Participants were assigned in a 1:1:1 ratio to one of the three ventilation strategies via a computer-generated random number table (block size = 6). An independent statistician who was not involved in patient recruitment, anesthesia administration, or data collection generated the random sequence using SPSS 26.0 software. Allocation sequences were enclosed in opaque, envelopes marked with sequential numbers, which were then stored in the hospital’s clinical trial office. It was only after the patient’s induction that the attending anesthesiologist accessed the corresponding envelope. Given the distinct designs of the oxygen delivery devices, blinding clinicians to group assignments was not possible; however, data analysts and outcome evaluators remained fully blinded to which group each patient belonged to.

### Standardized anesthesia protocol

When entering the operating room, immediately initiate monitoring of electrocardiogra m (ECG), bispectral index (Bis), oxygen saturation (SpO_2_), and mean arterial pressure (MAP). Prior to bronchoscopy, administer lidocaine (1%, 10 ml) via nebulization. All patients received standardized deep sedation with intravenous propofol and remifentanil, titrated to maintain BIS of 40–60. Spontaneous breathing was preserved unless rescue ventilation or intubation was required. All anesthesiologists had undergone formal training in the use of WNJ and prior to the study commencement. During anesthesia induction, oxygen is administered via an endoscopic mask. For induction, anesthesia was initiated via intravenous injection of propofol (1.5 mg/kg), remifentanil (1μg/kg); induction was considered complete once patients lost consciousness and was no response to verbal commands.

### Intervention protocols

**FIGURE 1:**
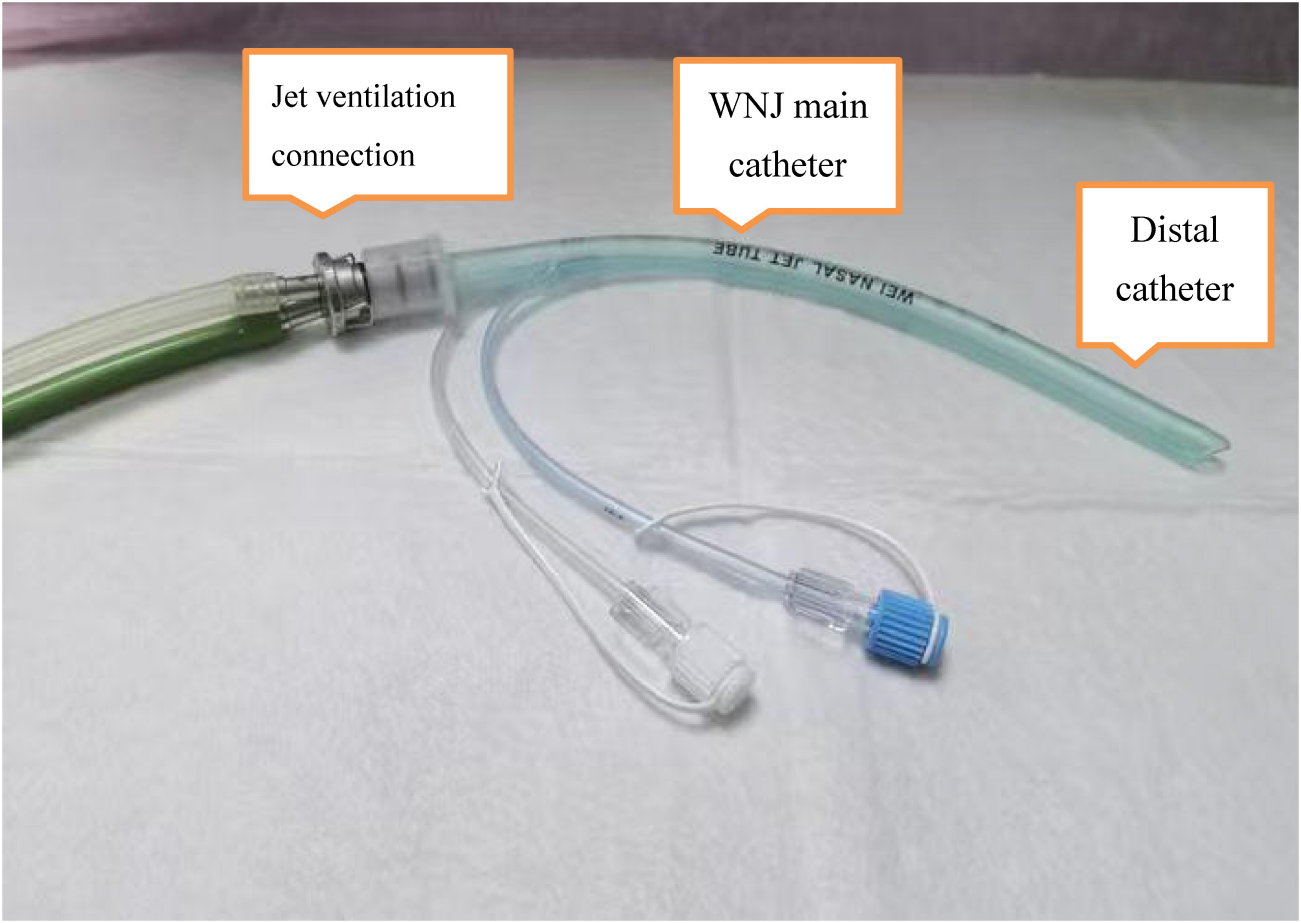
Schematic illustration of Wei nasal jet tube connected to the jet ventilator circuit. The Wei nasal jet tube was connected to the jet ventilator circuit and used to deliver supraglottic jet oxygenation and ventilation during bronchoscopic intervention under deep sedation. The figure illustrates the jet ventilation connection, WNJ main catheter, and distal catheter tip.

The outer part of the WNJ (Well Lead Medical Co., Ltd.) was coated with 5 mL of lidocaine ointment. The tube was inserted through one nostril and positioned approximately 1 cm above the vocal cords under electronic flexible bronchoscopic guidance. Then connected it to the ventilator (Figure 1)

If WNJ catheter insertion is unsuccessful the first time, another attempt may be performed via the opposite nasal cavity. If this second trial also fails, insertion of a WNJ catheter must be abandoned and an endoscopic mask used instead for ventilation. In cases of nasal bleeding during insertion, WNJ catheter insertion must be ceased immediately.

### Ventilation strategy

(1) COT group: Oxygen was administered via the proximal lateral port of the WNJ tube connected to anesthesia system (Mindray Bio-Medical Electronics Co., Ltd.). The flow was 10 L/min, fractional inspired oxygen (FiO_2_) 1.0.
(2) HFNC group: The tube was connected to High-Flow ventilator Device (Shenzhen Aobao Technology Co., Ltd., Shenzhen, China). Oxygen flow was initiated at 40 L/min with FiO_2_ of 1.0.
(3) SJOV group: Oxygen was delivered via the Twin Stream™ jet ventilator (Austria), with parameters set as follows: FiO_2_ at 1.0, Driving Pressure (DP) at 0.6 bar, Respiratory Rate (RR) at 600 cycles/min, and I/E ratio at 1:1.5.

### Oxygenation rescue algorithm

A standardized oxygenation safety algorithm was applied to all patients.

When SpO_2_ dropped below 95%, anesthesiologists shall perform jaw-thrust maneuver.

When SpO_2_ dropped below 90% the anesthesiologist shall adjust respiratory parameters for the three groups, respectively:

(1) COT group: Maximum oxygen flow rate set to 15 L/min;
(2) HFNC group: Maximum oxygen flow rate set to 80 L/min;
(3) SJOV group: Increase DP by 0.3 bar and RR by 300 cycles/min.

If SpO_2_ fell below 85%, the procedure must be immediately paused and the bronchoscope withdrawn. Subsequently, mask positive-pressure oxygenation (MPPO) or endotracheal intubation would be conducted if necessary. Resumption of bronchoscopy is permitted only when SpO_2_ reached ≥ 96%.

### Maintenance

For maintenance, propofol (2 mg/kg/h) and remifentanil (0.1 μg/kg/min) were administered continuously via intravenous infusion, with infusion rates adjusted to maintain hemodynamic stability [heart rate (HR) and blood pressure (BP)] varied within ± 30% of baseline; Bis: 40–60); ventilatory support was provided solely via the assigned oxygenation strategy.

Anesthesia was terminated at the conclusion of the procedure. Removal of WNJ was performed only when the patient is conscious, spontaneous breathing is fully restored, and SpO_2_ is ≥ 95%. If blood was observed during removal of the WNJ tube, local compression was applied until hemostasis was achieved.

### Data collection

The age, male, body mass index (BMI), past medical history of patients and procedure types were recorded by the data recorder.

The primary outcome was the rate of occurrence of hypoxemia (SpO_2_ < 90%), severe hypoxemia (SpO_2_ < 80%), and the necessity for rescue MPPO and intubation. The secondary outcomes were changes in arterial blood gas (ABG) parameters, and hemodynamic stability. The other outcomes were adverse events and procedure duration.

SpO_2_, MAP, and HR of patients were measured at baseline (T_0_), at the beginning of the procedure (T_*Beg*_), 15 min after the procedure started (T_15_), and at the conclusion of the procedure (T_*End*_). ABG analysis was performed at the standardized intraoperative time point of T_15_ to ensure consistency across groups and to minimize interference with the bronchoscopic procedure. Adverse events including hypotension (MAP < 30% of baseline), hypertension (MAP > 30% of baseline), tachycardia (HR > 30% of baseline), cough, body movement, sore throat, and nosebleed (blood observed during the removal of the WNJ tube) were recorded by data investigator. The procedure duration in the three groups was also recorded.

### Statistical analysis

This trial was designed with COT as the reference group and HFNC and SJOV as the two intervention groups. Owing to feasibility constraints and the anticipated recruitment during the study period, we intended to include 50 patients each group. With this sample size, the study provides approximately 63% power (two-sided α = 0.05) to detect a reduction in hypoxemia incidence from 30% in the COT group to 10% in an intervention group. Because the trial included two prespecified primary comparisons versus COT, statistical inference should be interpreted with consideration of multiplicity and the study’s feasible sample size. Continuous data are presented as mean ± SD. Categorical variables were presented using frequency counts and their corresponding percentages. A one-way ANOVA test was used to compare continuous variables across the three groups. Chi-square test was used for between-groups comparison of categorical data. Given the study’s sample size and dual primary comparisons, all statistical inferences should be interpreted cautiously, with consideration of multiplicity. All the statistical analyses were conducted using SPSS version 26.0.

## Results

**FIGURE 2:**
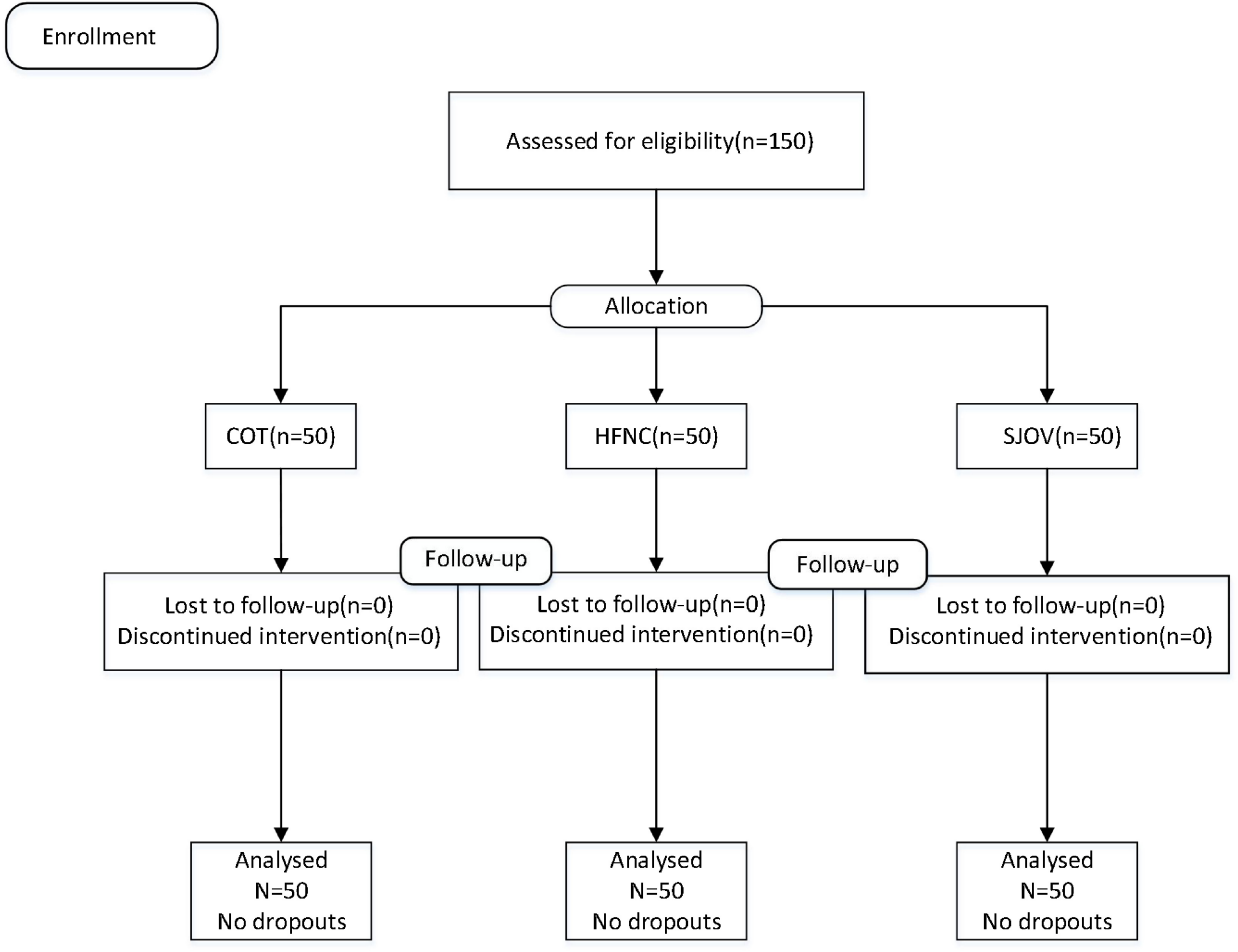
CONSORT flow diagram.

A qualification assessment was performed on 150 patients and they were randomly assigned to three groups (Figure 2). All randomized participants finished the study and were incorporated into the final analysis.

About the clinical data of patients, no significant statistical differences were observed in age, BMI, gender and procedure type among the three groups (Table 1).

**TABLE 1:** Comparison of preoperative patient characteristics among 3 groups.

Characteristics	COT (*n* = 50)	HFNC (*n* = 50)	SJOV (*n* = 50)	*P*-value
Age, y	52.1 ± 11.5	50.9 ± 12.2	50.9 ± 12.6	0.887
Male sex	32 (64.0)	31 (62.0)	33 (66.0)	0.905
BMI, kg⋅m^–2^	22.9 ± 3.0	23.0 ± 2.8	22.8 ± 2.9	0.909
Procedure type, (*n*, %)				0.978
Airway stenting	19 (38.0)	18 (36.0)	17 (34.0)	
Foreign body removal	13 (26.0)	15 (30.0)	16 (32.0)	
Biopsy	18 (36.0)	17 (34.0)	17 (34.0)	

Data were expressed as mean ± standard deviation or as numbers and percentages. COT: conventional-flow oxygen therapy; HFNC, High-flow nasal cannula; SJOV, supraglottic jet oxygenation and ventilation; BMI, body mass index.

### Primary outcomes

The number of cases with hypoxemia, severe hypoxemia, MPPO and endotracheal intubation were shown in Table 2. There was a significant difference in the overall incidence of hypoxemia across the three groups (*P* = 0.020). Pairwise comparison revealed that the incidence of hypoxemia in SJOV group was markedly lower compared to COT (*P* = 0.018), whereas no significant difference was found between SJOV and HFNC (*P* = 0.822) or COT and HFNC (*P* = 0.267). The incidence of severe hypoxemia varied significantly across the three groups (*P* = 0.024). Pairwise comparisons showed that SJOV had a significantly lower incidence of severe hypoxemia relative to the COT group (*P* = 0.016), though no significant difference was apparent among COT and HFNC (*P* = 0.362) or SJOV and HFNC (*P* = 0.719). MPPO incidence varied significantly among groups (*P* = 0.022). SJOV showed a lower rate than COT (*P* = 0.014), with no differences between SJOV and HFNC (*P* = 0.683) or COT and HFNC (*P* = 0.315). The incidence of endotracheal intubation was 0% in all three groups, no notable differences were detected between the groups.

**TABLE 2:** Primary outcomes.

Patients values (*n*, %)	COT (*n* = 50)	HFNC (*n* = 50)	SJOV (*n* = 50)	*P*-value
Hypoxemia	15 (30.0%)^b^	8 (16.0%)	5 (10.0%)^a^	0.020
Severe hypoxemia	9 (18.0%)^b^	4 (8.0%)	1 (2.0%)^a^	0.024
MPPO	11 (22.0%)^b^	6 (12.0%)	2 (4.0%)^a^	0.022
Endotracheal intubation	0 (0.0%)	0 (0.0%)	0 (0.0%)	1.000

Data were presented as numbers and percentages. a was statistically significant compared with COT group, b was statistically significant compared with the SJOV group, c was statistically significant compared with the HFNC group. COT, conventional-flow oxygen therapy; HFNC, High-flow nasal cannula; SJOV, supraglottic jet oxygenation and ventilation; MPPO, mask positive pressure oxygenation.

### Secondary outcomes

Arterial blood gas parameters are shown in Table 3. The results of PaO_2_ and lactic acid showed significant differences across the three groups (*P* < 0.001 and *P* < 0.001, individually). Post hoc pairwise comparisons revealed that PaO_2_ was significantly higher in the SJOV group than in both the COT and HFNC groups (*P* < 0.001 and *P* < 0.001, respectively), whereas no notable difference was detected between the COT and HFNC groups. Lactic acid was significantly lower in SJOV relative to COT (*P* < 0.001) and HFNC (*P* = 0.006) groups, and was also lower in the HFNC group than in the COT group (*P* < 0.001). No notable differences in PaCO_2_ or pH were observed across the three groups (*P* = 0.425 and 0.352, respectively).

**TABLE 3:** Secondary outcomes.

Patients values mean ± SD	COT (*n* = 50)	HFNC (*n* = 50)	SJOV (*n* = 50)	*P*-value
PaO_2_ (mmHg)	72.3 ± 10.5^c^	68.9 ± 9.8^c^	128.5 ± 15.2^ab^	< 0.001
PaCO_2_(mmHg)	38.5 ± 4.2	39.2 ± 3.9	37.8 ± 4.0	0.425
pH	7.38 ± 0.03	7.37 ± 0.04	7.39 ± 0.03	0.352
Lactic acid (mmol/l)	2.0 ± 0.4^bc^	1.6 ± 0.3^ac^	1.1 ± 0.3^ab^	< 0.001

Data were presented as mean ± standard deviation (median, range). a was statistically significant compared with the COT group, b was statistically significant compared with the HFNC group, c was statistically significant compared with the SJOV group. COT, conventional-flow oxygen therapy; HFNC, High-flow nasal cannula; SJOV, supraglottic jet oxygenation and ventilation; PaO_2_, partial pressure of oxygen; PaCO_2_, partial pressure of arterial carbon dioxide; Ph, potential of Hydrogen.

### Other outcomes

Perioperative adverse events and procedure duration were summarized in Table 4. Overall, there was no notable difference in the occurrence of adverse events among three groups. Specifically, hypotension emerged in 6 (12.0%) patients in COT group, 5 (10.0%) patients in HFNC group, and 2 (4.0%) patients in SJOV group (*P* = 0.352). Hypertension occurred in 3 (6.0%) patients in COT group, 2 (4.0%) patients in HFNC group, and 1 (2.0%) patient in SJOV group (*P* = 0.538). Tachycardia was reported in 5 (10.0%), 4 (8.0%), and 2 (4.0%) patients, respectively (*P* = 0.641). Other events, including cough, body movement, sore throat, and nosebleed, were comparable across groups (*P* > 0.05, respectively). The average duration of the procedure was comparable across the groups: 28.0 ± 6.3 min in the COT group, 27.7 ± 5.8 min in the HFNC group, and 27.1 ± 5.5 min in SJOV (*P* = 0.712). There were no significant differences in total propofol or remifentanil consumption among the three ventilation groups. The total propofol consumption was 199.4 ± 32.0 mg in the COT group, 200.1 ± 38.9 mg in the HFNC group, and 192.6 ± 40.2 mg in the SJOV group (*P* = 0.508). The total remifentanil consumption was 232.5 ± 73.4 μg, 233.3 ± 73.8 μg, and 240.7 ± 91.0 μg in the COT, HFNC, and SJOV groups, respectively (*P* = 0.995).

**TABLE 4:** Other outcomes.

Adverse event	COT (*n* = 50)	HFNC (*n* = 50)	SJOV (*n* = 50)	*P*-value
Hypotension (*n*, %)	6 (12.0)	5 (10.0)	2 (4.0)	0.352
Hypertension (*n*, %)	3 (6.0)	2 (4.0)	1 (2.0)	0.538
Tachycardia (*n*, %)	5 (10.0)	4 (8.0)	2 (4.0)	0.641
Cough (*n*, %)	4 (8.0)	3 (6.0)	5 (10.0)	0.712
Body movement (*n*, %)	6 (12.0)	5 (10.0)	3 (6.0)	0.538
Sore throat (*n*, %)	3 (6.0)	4 (8.0)	4 (8.0)	0.894
Nosebleed (*n*, %)	2 (4.0)	2 (4.0)	3 (6.0)	0.865
Procedure duration (min)	28.0 ± 6.3	27.7 ± 5.8	27.1 ± 5.5	0.712
Total propofol consumption (mg)	199.4 ± 32.0	200.1 ± 38.9	192.6 ± 40.2	0.508
Total remifentanil consumption (μg)	232.5 ± 73.4	233.3 ± 73.8	240.7 ± 91.0	0.995

There were no significant differences among the three groups in adverse events, procedure duration, total propofol consumption, or total remifentanil consumption. COT, conventional-flow oxygen therapy; HFNC, high-flow nasal cannula; SJOV, supraglottic jet oxygenation and ventilation.

**FIGURE 3:**
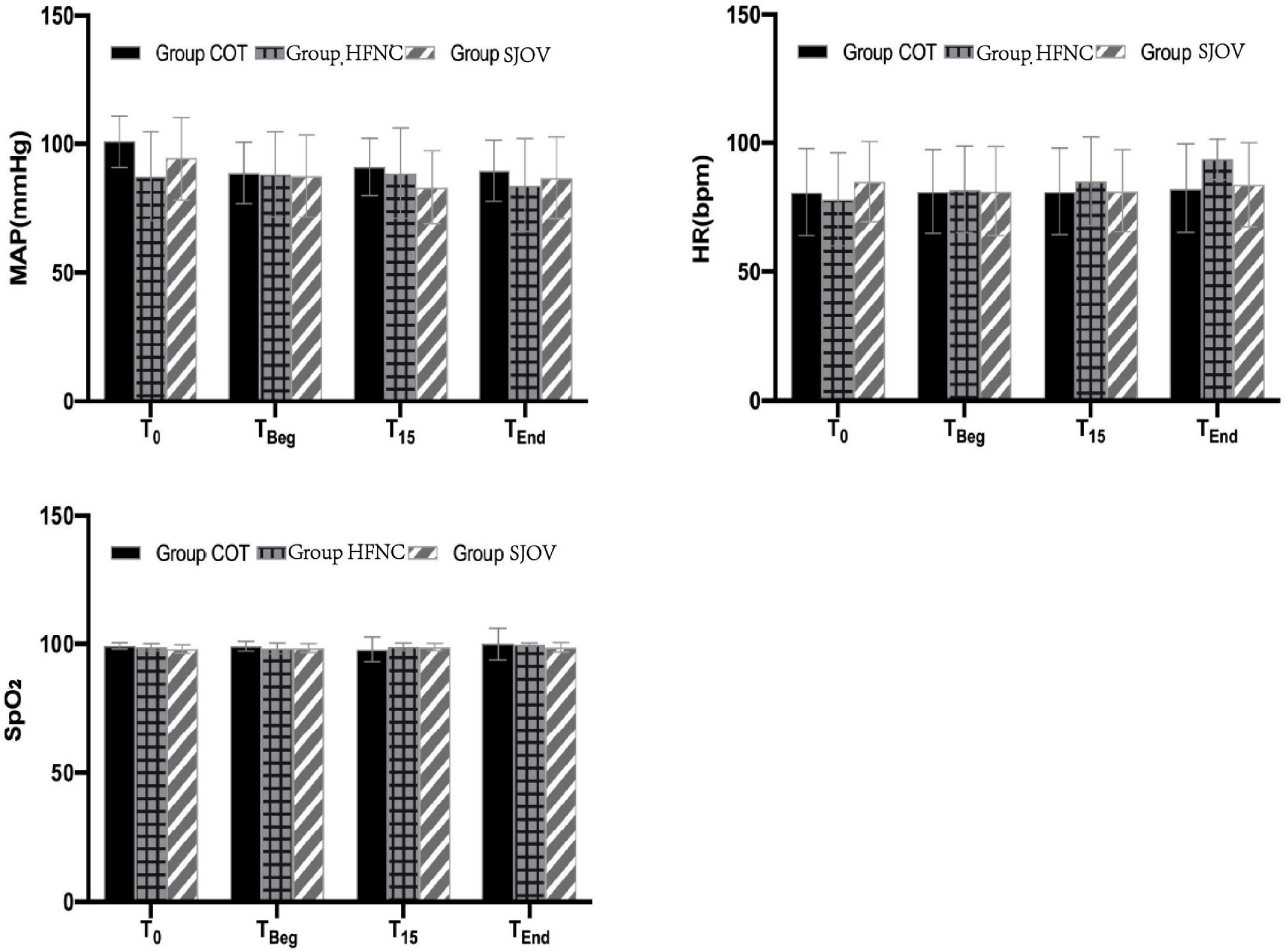
Hemodynamic indexes among three groups. No significant differences among three groups in MAP, HR and SpO_2_ at different time points. Patients’ SpO_2_, MAP and HR were recorded at baseline (T_0_), beginning of procedure (T_*Beg*_), 15 min after initiation of procedure (T_15_), and at the end of procedure (T_*End*_). COT, conventional-flow oxygen therapy; HFNC, high-flow nasal cannula; SJOV, supraglottic jet oxygenation and ventilation; SpO_2_, pulse oxygen saturation; MAP, mean arterial pressure; HR: heart rate.

For MAP, values in all groups remained within the normal range at each time point. At T_0_, the MAP in the COT group was slightly higher than that in the HFNC and SJOV groups; however, the difference was not statistically significant (*P* = 0.312). Similarly, no significant intergroup differences were observed at T_*Beg*_, T_15_, or T_*End*_ (*P* = 0.428, *P* = 0.367, and *P* = 0.401, respectively). Regarding HR, HR remained generally stable in all groups throughout the study period. No statistically significant differences were found among the three groups at T_0_, T_*Beg*_, T_15_, or T_*End*_ (*P* = 0.276, *P* = 0.338, *P* = 0.291, and *P* = 0.184, respectively). For SpO_2_,comparisons among the three groups at each time point revealed no statistically significant differences (T_0_: *P* = 0.517; TBeg: *P* = 0.463; T15: *P* = 0.389; T_*End*_: *P* = 0.441). Overall, between-group comparisons showed no significant differences in MAP, HR, or SpO_2_ at any recorded time point (all *P* > 0.05; Figure 3).

## Discussion

In recent years, BI has made significant advances in the field of pulmonary medical diagnosis and treatment. Monitored Anesthesia Care (MAC) is administered during BI (10). By maintaining the breathing spontaneous without laryngeal mask airway or endotracheal intubation, the MAC anesthesia is less painful while fulfilling the surgical needs (11). The most frequently employed ventilation modes were COT (10, 12). Despite the utility, procedural hypoxemia remains the most frequent and hazardous complication, with reported incidence ranging from 30% to as high as 70% (13).

Recently, the use of HFNC during advanced bronchoscopy has garnered increasing attention (14). It can improve CO_2_ clearance efficiency and reduce respiratory workload burden (15). SJOV provides high-frequency, high-pressure oxygen pulses through a catheter placed above the glottis, which effectively flushes pharyngeal dead space and generates functional PEEP without obstructing the endoscopic field, thus theoretically addressing the key limitations of COT (16–18).

This randomized controlled trial involving 150 patients systematically evaluated and compared the ventilatory efficacy of COT, HFNC, and SJOV via WNJ under MAC. Our findings demonstrated that the SJOV group exhibited the lowest incidence rates of hypoxemia, severe hypoxemia and MPPO, whereas COT group showed the highest incidence. This difference may be attributed to the different mechanisms of each type of ventilation or oxygenation therapy. SJOV had been shown to limit alveolar overdistension and atelectasis, thereby preserving lung compliance and improving ventilation–perfusion (V/Q) matching. These protective effects are largely attributable to the delivery of very small tidal volumes at high frequencies with passive expiration. This ventilation pattern substantially reduces peak and mean airway pressures, which are commonly accepted surrogate indicators of alveolar stress, while facilitating effective intrapulmonary gas mixing (6, 19). In a simulated open airway rigid bronchoscope model, SJOV shows this characteristic mechanical behavior. With the increase of ventilation frequency, the peak airway pressure decreases, the PEEP gradually increases, and the amount of tidal gas decreases significantly. At a driving pressure of 0.3 bar and a frequency of 300 times per minute, the peak airway pressure generated by SJOV is 4.5–4.7 cmH_2_O, the PEEP is 0.8 cmH_2_O, and the tidal volume is about 24 mL, which supports its ability to minimize the excessive expansion pressure leading to ventilator-related lung injury. In addition to low pressure, the per-minute ventilation volume is also sufficient to meet the oxygenation demand (20). Consistent with these physiological findings, clinical evidence shows that SJOV can maintain sufficient oxygenation at lower airway pressure than traditional ventilation strategies for patients undergoing shared airway surgery or impaired respiratory function (21, 22).

The difference in the main results is further reflected in the secondary results, especially the ABG results. SJOV produces significantly higher PaO_2_ values, which is consistent with the physiological principle of SJOV producing functional PEEP to better maintain arterial oxygenation. The improvement of oxygenation is attributed to the minute ventilation generated by the airflow (23). In contrast, when the bronchoscope occupies the airway cavity, the PEEP effect of HFNC is significantly reduced, while COT cannot provide measurable expansion pressure, which explains the reason for their poor oxygenation ability (24).

There was a significant difference in the incidence of hypoxemia and PaO_2_ among the study groups, while there was no significant difference in PaCO_2_ or pH. The absence of hypercapnia or acidosis may be attributed to the relatively short time of bronchoscopic intervention and the exclusion of patients with severe airway obstruction, both of which are conducive to maintaining the exhalation rate and effectively removing carbon dioxide. In the case of airway narrowing, SJOV can improve the removal of CO_2_ by increasing the effective minute ventilation (25, 26). Continuous dynamic carbon dioxide monitoring was not performed in the present study; however, arterial blood gas analysis obtained at the standardized intraoperative time point did not demonstrate significant hypercapnia among groups. Patients undergoing prolonged intervention or those with impaired baseline ventilation may still be at risk of CO_2_ retention, which warrants further investigation.

The lactic acid level of the SJOV group is the lowest, reflecting the reduction of the overall oxygen debt. The higher PaO_2_ and improved V/Q coupling achieved by SJOV weaken anaerobic metabolism, reduce respiratory muscle load, and inhibit the release of catecholamine, thus reducing the production of cellular lactic acid. This metabolic advantage means better tissue perfusion and may translate into a reduction in cardiovascular load in high-risk patients (27, 28).

It is worth noting that the hemodynamics of these three groups of subjects remained stable, which can be attributed to two factors: (1) The standardized sedation regimen uses low doses of propofol and remifentanil to minimize cardiovascular inhibition (29); (2) The dual-cavity design of WNJ avoids stimulation of the larynx during ventilation, thus reducing hemodynamic fluctuations.

This study found that there was no significant difference in adverse events between the three treatment regimens of COT, HFNC and SJOV, which was partially consistent with the results of a multicenter trial (30). The study showed that among 600 subjects, the incidence of sore throat (8.2, 7.9 and 8.5%, respectively) and the incidence of nosebleeds (3.1, 2.8 and 3.3%, respectively) of the three treatment methods were comparable. In the SJOV group, the incidence of hypotension, tachycardia and body movement was lower than that of the COT group and the HFNC group, but the difference was not statistically significant, indicating that SJOV did not increase hemodynamic fluctuations or airway damage. The incidence of nosebleeds and sore throats in the three treatment groups is also similar, indicating that the WNJ catheter has good tolerance (31). Overall, SJOV significantly improves oxygenation and does not introduce additional risks, and its safety is comparable to that of traditional oxygen therapy strategies.

### Limitations

Several limitations should be acknowledged. First, this was a single-center study conducted in a tertiary referral center with experienced bronchoscopic and anesthetic teams, which may limit the external generalizability of the findings. Second, due to the obvious physical differences among oxygenation devices, anesthesiologists and procedural operators could not be completely blinded to group allocation. Nevertheless, the main respiratory outcomes were predefined and objectively measured, while outcome assessors and data analysts remained blinded. Third, only patients with mild-to-moderate airway stenosis were included, which limits the applicability of the findings to high-risk patients with severe airway obstruction or severe baseline respiratory compromise. Fourth, although procedure types were balanced across groups, the present study was not specifically powered to evaluate procedure-specific respiratory outcomes. Fifth, arterial blood gas measurements were obtained at a standardized intraoperative time point, and serial arterial blood gas sampling or continuous CO_2_ trend monitoring was not incorporated into the current study design. Finally, postoperative recovery quality, patient-reported outcomes, and long-term clinical outcomes were not evaluated. Future multicenter studies with larger sample sizes are warranted to validate these findings in broader and higher-risk patient populations.

## Conclusion

In selected patients with mild-to-moderate airway stenosis undergoing bronchoscopic intervention under deep sedation, SJOV delivered via the WNJ improved arterial oxygenation and reduced hypoxemia compared with COT, without increasing adverse events. Further multicenter studies are needed to confirm its efficacy and safety in broader and higher-risk populations.

## Data Availability

The original contributions presented in this study are included in this article/supplementary material, further inquiries can be directed to the corresponding authors.
